# A short review on guided implant surgery and its efficiency

**DOI:** 10.6026/97320630018764

**Published:** 2022-09-30

**Authors:** B. Vyshnavi Sindhusha, Arvina Rajasekar

**Affiliations:** 1 Department of Periodontics, Saveetha Dental College and Hospitals, Saveetha Institute of Medical & Technical Sciences, Chennai- 600077, Tamilnadu, India

**Keywords:** Dental implants, Virtual plan, Computer-assisted surgery

## Abstract

Ideal implant placement may reduce surgical complications, such as nerve injury and lingual cortical plate perforation, and minimize the likelihood of functional and prosthetic compromises. Guided implant surgery [GIS] has been used as the means to achieve ideal implant placement. GIS refers to the process of digital planning, custom-guide fabrication, and implant placement using the custom guide and an implant system–specific guided surgery kit. GIS includes numerous additional steps beyond the initial prosthetic diagnosis, treatment planning, and fabrication of surgical guides. Substantial errors can occur at each of these individual steps and can accumulate, significantly impacting the final accuracy of the process with potentially disastrous deviations from proper implant placement. Pertinent overall strategies to reduce or eliminate these risks can be summarized as follows: complete understanding of the possible risks is fundamental; knowledge of the systems and tools used is essential; consistent verification of both diagnostic and surgical procedures after each step is crucial; proper training and surgical experience are critical. This review article summarizes information on the accuracy and efficacy of GIS, provides insight on the potential risks and problems associated with each procedural step, and offers clinically relevant recommendations to minimize or eliminate these risks.

## Background:

The placement of osseointegrated dental implants is nowadays a common procedure in periodontal clinical practice. Implant therapy is driven by the patients' restorative needs and steered by the esthetic and functional demands of each case; concurrently, implant therapy can be limited by anatomic constraints. Therefore, correct implant positioning is critical if an esthetically and functionally acceptable restoration that can be maintained through proper oral hygiene is to be achieved. Furthermore, implant placement must respect the various critical anatomic elements often present in the vicinity of a restoratively dictated implant site. Consequently, during diagnosis and treatment planning, the implant surgeon must pay close attention to both restorative and anatomic restrictions while selecting an alveolar bone site of adequate quality and quantity to ensure both appropriate and safe implant placement. Implant therapy diagnosis and treatment planning during the early years of osseointegration was based on clinical examination, study casts, and radiographs (periapical and panoramic). The inherent limitations of these two-dimensional radiographic techniques were overcome with the adoption of digital computed tomography, and, subsequently, the more widespread use of dental cone beam computed tomography that has allowed the detailed and precise three-dimensional evaluation of osseous topography [1]. Guided implant surgery refers to the process of digital planning, custom-guide fabrication, and implant placement using the custom guide and an implant system-specific guided surgery kit. It is evident from the aforementioned description that guided implant surgery includes numerous steps beyond the initial prosthetic diagnosis and treatment planning and fabrication of prosthetic radiographic guide: patient scanning, conversion of digital imaging files and importation into a software program, completion of virtual implant treatment planning, integration of a virtual model of the teeth (derived from an intraoral surface scan or an extraoral scan of stone casts) with the cone beam computed tomography model, selection and fabrication of surgical guide, and implant placement using the fabricated surgical guide [2]. In cases of immediate loading, the process typically also includes connection of the prepared provisional restoration to the implant before the patient visit is completed. The preparation of the osteotomy during static surgery is aided by patient-specific surgical guides created to transmit the virtual. Implant placement to the patient and re-create the perfect implant placement in terms of position, angle and depth [3-10]. Surgical guides can be made with the help of models, fast prototyping, or stereolithographic technologies. Model-based guides are milled or digitally printed in the lab or processed utilizing computer aided design/computer-assisted manufacturing. Photopolymerization techniques based on three-dimensional imagery and design are used to create stereolithographic guides. This review aims to summarize information on the accuracy and efficacy of static guided implant surgery with special emphasis on the risks and potential problems of every step in the process. In addition, recommendations and procedures to prevent or eliminate these risks will be addressed.

## Steps in guided implant surgery:

[1] Patient scanning or initiator prosthetic diagnosis for the placement of the implant in the desired position [11].

[2] Conversion of the digital imaging files and importation of the digital images into the software program.

[3] Completion of virtual implant treatment planning.

[4] Integration of a virtual model of the teeth with the cone beam computed tomography model [derived from an intraoral surface scan or an extraoral scan of stone casts [13].

[5] Selection and fabrication of surgical guide based on the computed tomography model.

[6] Implant placement using the fabricated surgical guide.

## Advantages:

[1] Involvement of all dental care providers from beginning, thus ensuring comprehensive diagnosis and treatment planning and better outcomes.

[2] Serious potential complications of implant surgery that can be minimized by guided implant surgery- such as injury to the critical anatomical structures like sinus, nerves, vessels and teeth [13-15].

[3] Allows flapless implant placement [19], which reduces crestal bone resorption associated with flap elevation [20-22].

[4] Better maintenance of the soft tissue profiles -Gingival margin position, interdental papilla.

[5] From practice management point- guided implant surgery offers faster and more accessible record keeping and filing.

[6] Avoidance of flap elevation and sutures results in less postoperative pain, edema, and bleeding, and immediate resumption of oral hygiene procedures [23-26].

[7] Evidence suggests that putting an implant through the gingiva has no effect on osseointegration, bone levels, or aesthetic outcomes, as long as the soft tissue punch employed is not greater than the implant diameter [27-28].

[8] When flap elevation and sutures are avoided, postoperative pain, edema, and bleeding are reduced, and oral hygiene practices can be resumed right away [29]. In contrast, even for experienced surgeons, flapless implant placement without guided implant surgery can pose considerable hazards [30].

## Disadvantages:

[1] Increased preoperative treatment planning time and additional cost of guide fabrication for each case.

[2] Initial cost for the purchase of adequate computer hardware and software programs and special instructions and drills are expensive [10].

[3] Additional training and familiarization with software will take a bit of time.

[4] The routine and correct use of guided implant surgery demands a change in philosophy regarding implant placement and necessitates a learning curve and expenses related to training and familiarization with the software and the tools provided [5,8]; such a change in practice philosophy can represent a real challenge for an established practitioner.

## Guided implant surgery outcomes compared to conventional implant placement:

In totally edentulous patients, the successful use of guided implant surgery combined with the simultaneous delivery of a prefabricated prosthesis for the rapid replacement of missing teeth has been described.

The use of guided implant surgery in partially edentulous patients has also been evaluated; out of 250 implants (102 patients), 58% were placed flapless, one planned implant had to be changed to a shorter one, and in four posterior cases the limited interocclusal distance presented challenges during drilling; in eight cases, implant placement had to be delayed (guides could not be used) because of required bone augmentation, and for nine of the implants the final angle differed from the planned one, without any resulting clinical consequences. When guided implant surgery with bone supported guides (n = 16) or mucosa supported guides (flapless, n = 15) was directly compared with the conventional implant placement protocol (n = 21), flapless procedures lasted significantly less time and resulted in less postoperative pain, analgesic consumption, and instances of trismus and of patient reported hemorrhage [31-34].However, none of the mentioned studies were randomized controlled trials. In recent years, a few randomized controlled trials have compared guided implant surgery with conventional implant placement [35-38].

The available randomized controlled trials suggest that guided implant surgery results in greater accuracy, less post operative pain or pain of shorter duration, less swelling, and less surgery time (guided implant surgery system dependent), but at higher financial cost; there were no differences between guided implant surgery and conventional surgery in terms of implant success/failure or clinical parameters such as marginal bone loss at 1 year [39].

## Associated risks and prevention:

### Risks:

[1] Poor image quality and dimensional positional accuracies in the cone beam computerized tomography [35-38].

[2] Improper or inadequate virtual implant positioning in treatment planning software.

[3] Improper fit of the surgical guide or the surgical guide fracture [39].

### Prevention:

[1] Use of occlusal bite index to stabilize lower jaw and prosthesis- to improve image quality [40].

[2] Knowledge of limitations and accuracy level of specific software - to correct the virtual implant positioning [43].

[3] Use appropriate fixation such as three mini screws to stabilize mucosa or bone-supported guides along with special attention when using bone-supported guides [44-45].

## Clinical implications:

When using guided implant surgery the most important inaccuracy is in the vertical dimension (osteotomy depth), with inaccuracy in mesio-distal or bucco-lingual direction being clearly less. This is possibly due to the presence of debris in the implant cavity so that the implant cannot reach its final position, the resilience of the mucosal tissues in mucosa-supported guides, the setting of the gray values during segmentation, the blockage of the implant holders in the sleeves or by the crestal bone, and the deformation of the guide during surgery [45-47].

## Conclusions:

Guided implant surgery can be an accurate and clinically advantageous procedure when implant therapy is called for. However, substantial errors can occur at each individual step and can accumulate, significantly impacting the final accuracy of the process with potentially disastrous deviations from ideal implant placement. It is possible to eliminate or reduce these risks, provided that complete understanding of the guided implant surgery process, thorough and careful surgical technique, advanced comprehensive training, and adequate case preparation are always in place.
